# Improvement in the photoelectrochemical responses of PCBM/TiO_2 _electrode by electron irradiation

**DOI:** 10.1186/1556-276X-7-142

**Published:** 2012-02-20

**Authors:** Seung Hwa Yoo, Jong Min Kum, Ghafar Ali, Sung Hwan Heo, Sung Oh Cho

**Affiliations:** 1 Department of Nuclear and Quantum Engineering, Korea Advanced Institute of Science and Technology, Daejeon, 305-701, South Korea; 2 Nanomaterials Research Group, Physics Division, PINSTECH, Islamabad, 45650, Pakistan; 3 Particla Co. Ltd., Daejeon, 306-220, South Korea

**Keywords:** photoelectrochemical cell, TiO_2_, electron irradiation, PCBM, band-edge tuning

## Abstract

The photoelectrochemical (PEC) responses of electron-irradiated [6,6]-phenyl-C61-butyric acid methyl ester (PCBM)/TiO_2 _electrodes were evaluated in a PEC cell. By coating PCBM on TiO_2 _nanoparticle film, the light absorption of PCBM/TiO_2 _electrode has expanded to the visible light region and improved the PEC responses compared to bare TiO_2 _electrode. The PEC responses were further improved by irradiating an electron beam on PCBM/TiO_2 _electrodes. Compared to non-irradiated PCBM/TiO_2 _electrodes, electron irradiation increased the photocurrent density and the open-circuit potential of PEC cells by approximately 90% and approximately 36%, respectively at an optimum electron irradiation condition. The PEC responses are carefully evaluated correlating with the optical and electronic properties of electron-irradiated PCBM/TiO_2 _electrodes.

## Introduction

TiO_2 _has been widely used for photocatalysts because of its good chemical- and photostabilities to convert photon energy to electrical and chemical energies [1]. However, due to its wide bandgap, the light absorption is limited only to the ultraviolet (UV) region of the solar spectrum. Hence, sensitizing TiO_2 _with small bandgap semiconductors, such as quantum dots or organic dyes, has been extensively studied to harvest more photons in the visible light region of solar spectrum for the applications to quantum dot-sensitized solar cells [2-4], dye-sensitized solar cells [5-7], and photoelectrochemical (PEC) cells [8-10].

Along with this current research trends, combining TiO_2 _with carbonaceous nanomaterials has attracted much interest, and studies on these materials are increasing exponentially these days [11]. For instance, high performance photocatalysts such as carbon nanotube-TiO_2 _[12-14], fullerene-TiO_2 _(C_60_-TiO_2_) [15-17], and graphene-TiO_2 _[18,19] composites have been introduced by several groups and have shown enhanced photocatalytic activities. Notably, C_60 _has shown interesting effects when combined with TiO_2_: facilitating the separation of photo-generated charge carriers from TiO_2 _to C_60 _[15,16] or sensitizing TiO_2 _to absorb visible light [17]. However, the band-edge position of C_60 _is unfavorable for a sensitizer of TiO_2 _because the lowest unoccupied molecular orbital (LUMO) level of C_60 _is lower than the conduction band of TiO_2 _[17]. From the viewpoint of energy levels, [6,6]-phenyl-C61-butyric acid methyl ester (PCBM) is a better candidate than C_60 _for the sensitization of TiO_2_. We expect that the photo-excited electrons of PCBM can be transferred to TiO_2 _more efficiently because the LUMO level of PCBM is slightly higher than the conduction band of TiO_2 _[20]. In our previous study, we have found that the band-edge positions as well as the bandgap of PCBM can be tuned by electron irradiation at different fluences [21]. We believe that electron irradiation technique can be an alternative and unique method to modify the molecular structure and tune the bandgap [22,23] compared to the conventional methods such as adjusting the particle size of quantum dots [24,25] or modifying the molecular structure of the dyes [26] for larger light absorption. In addition to the bandgap, the band-edge positions can also be tuned by electron irradiation compared to the conventional methods such as ionic adsorption for specific quantum dots [27] or by varying the conjugation linkers in organic dyes [28].

Based on our previous findings, we present here a novel approach to improve the PEC performance of PCBM/TiO_2 _electrodes using electron beam irradiation. The photocurrent density and open-circuit potential of PCBM/TiO_2 _were respectively improved by 90% and 36% by electron irradiation. The effects of the electron irradiation on the PEC performances of PCBM/TiO_2 _were systematically analyzed in this study.

## Methods

**Figure 1:**
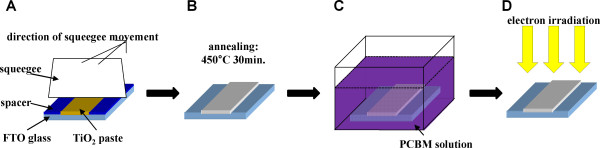
**Schematic representation of the preparation of PCBM/TiO_2 _electrode and subsequent electron irradiation**. (**A**) Deposition of TiO_2 _paste by doctor blade technique. (**B**) Formation of TiO_2 _nanoparticle film by annealing the as-deposited TiO_2 _paste at 450°C for 30 min. (**C**) Fabrication of PCBM/TiO_2 _electrode by immersing TiO_2 _electrode in 1.5 mM PCBM solution for 5 h. (**D**) Electron irradiation on PCBM/TiO_2 _electrode at different fluences.

Figure 1 shows the schematic representation of the preparation of PCBM/TiO_2 _electrode and subsequent electron irradiation. The as-received TiO_2 _nanoparticle paste (DSL 18NR-T, Dyesol Industries Pty Ltd., Queanbeyan, New South Wales, Australia) was deposited on a fluorine-doped tin oxide (FTO) glass substrate (8 Ωm^-2^, Dyesol) by a doctor blade technique. Before the deposition of TiO_2 _paste, FTO glass substrates were cut by 1.0 × 2.5 cm^2 ^in dimension and were sonicated successively in acetone, isopropanol, ethanol, and deionized water for thorough cleaning and dried in N_2 _gas stream. After the deposition of TiO_2 _paste, subsequent annealing process was performed at 450°C for 30 min with a temperature increase rate of 1°C min^-1^. After the annealing, TiO_2 _nanoparticle film was formed. The as-prepared TiO_2 _electrodes were immersed vertically in a chlorobenzene solution containing 1.5 mM PCBM for 5 h while stirring. PCBM solution was prepared by dissolving PCBM (99.5% purity, Nano-C, Inc., Westwood, MA, USA) powder into chlorobenzene (≥99.5% purity, Sigma-Aldrich, St. Luois, MO, USA) solvent. After the immersion, the electrodes were washed in pure chlorobenzene several times and dried at ambient condition. As a result, PCBM/TiO_2 _electrodes, where a thin layer of PCBM was coated on the TiO_2 _nanoparticle electrodes, were prepared. Coating process of PCBM was carried out in darkness. The irradiation of an electron beam on PCBM/TiO_2 _electrodes was carried out at room temperature and in vacuum lower than 2 × 10^-5 ^Torr. An electron beam was generated from a thermionic electron gun with electron energy of 50 keV, and current density of the electron beam was 1.6 μA cm^-2^. The electron fluence was varied by adjusting the irradiation time. PCBM/TiO_2 _electrodes were irradiated by 1, 2, and 4 h which correspond to electron fluence of 3.6 × 10^16^, 7.2 × 10^16^, and 1.44 × 10^17 ^cm^-2^, respectively. Diffuse reflectance UV-visible (VIS) spectra of electron-irradiated PCBM/TiO_2 _powders were measured on a spectrometer (S-4100, SCINCO CO., LTD., Seoul, South Korea) by scratching the nanoparticle film off the FTO glass substrate.

After electron irradiation of PCBM/TiO_2 _electrodes, a custom-made PEC cell was constructed to measure the PEC responses of electron-irradiated PCBM/TiO_2 _electrodes, which act as photo-anodes of PEC cells. The PEC cell has a three-electrode configuration comprising a photo-anode, a Pt wire as a cathode, and a saturated calomel electrode (SCE) (0.242 V vs. NHE, BAS Inc., West Lafayette, IN, USA) as a reference electrode. An aqueous solution of 1 M NaOH (Junsei Chemical Co., Ltd., Chuo-ku, Tokyo, Japan) was used as a supporting electrolyte after 30 min purging with N_2 _gas. The PEC response of the electrodes was recorded on a potentiostat (Model SP-50, BioLogic, Claix, France) by sweeping the potential from -1.2 to 0.5 V (vs. SCE) at a sweep rate of 100 mV s^-1^. The photo-anodes were illuminated with a solar simulator (Model LS-150, Abet Technologies, Inc., Milford, CT, USA) equipped with AM 1.5 filter. The illumination power was estimated as 80 mW cm^-2 ^at the photo-anode surface by a digital photometer (ILT1400-A, International Light Technologies, Inc., Peabody, MA, USA).

## Results and discussion

**Figure 2:**
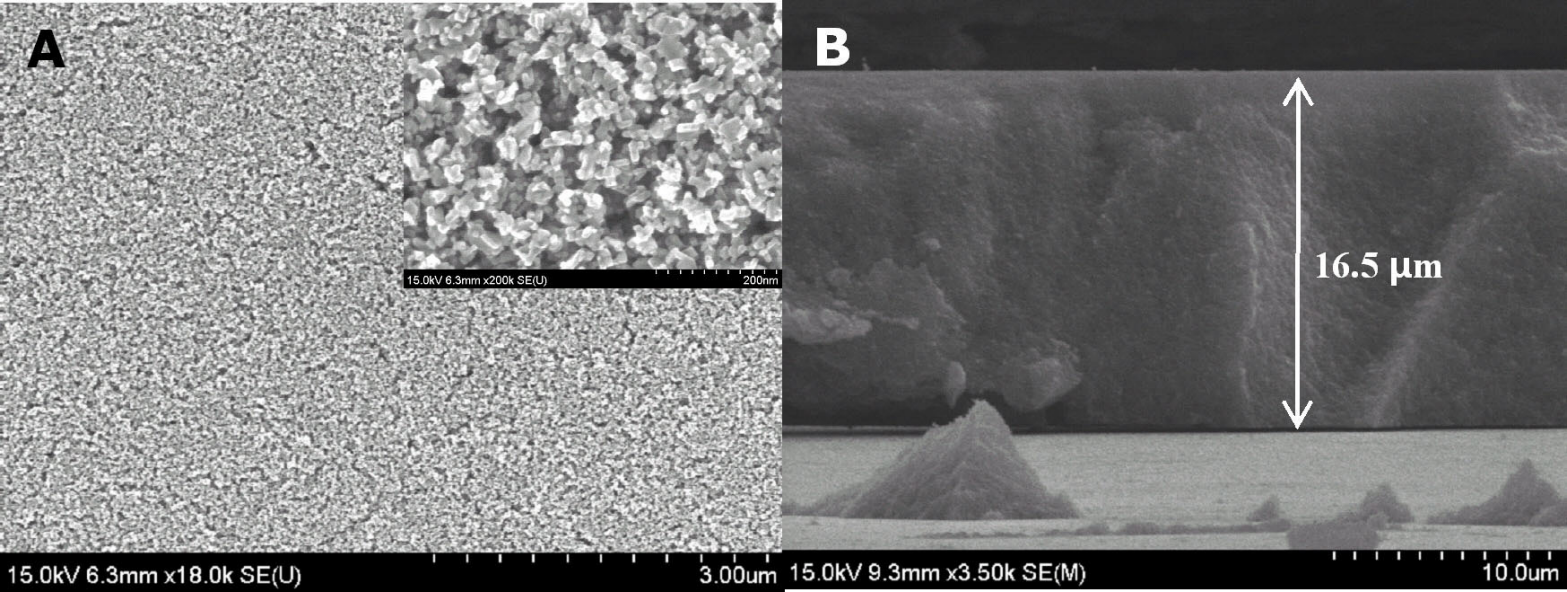
**FESEM images**. (**A**) Top view and higher magnification (inset) and (**B**) cross-sectional view of TiO_2 _nanoparticle film.

Figure 1 displays the schematic representation for the preparation of the PCBM/TiO_2 _photo-anodes of PEC cells. TiO_2 _nanoparticles (NPs) were firstly deposited to form a film on a FTO glass substrate. A uniform TiO_2 _NP film was formed by annealing the as-deposited TiO_2 _paste at 450°C for 30 min. The TiO_2 _NP film was submerged in a PCBM solution for 5 h, and consequently, the TiO_2 _NP film was coated with PCBM. Subsequently, the PCBM/TiO_2 _electrodes were irradiated with an electron beam. The energy of the electron beam was 50 keV, and the electron fluence was changed by controlling the irradiation time. These electron-irradiated PCBM/TiO_2 _films on FTO glass substrates were used as photo-anodes of PEC cells for water splitting. Figure 2 shows the field emission scanning electron microscopy (FESEM) images of the fabricated PCBM/TiO_2 _film. TiO_2 _NPs with the diameter of approximately 20 nm were deposited on a FTO glass substrate (see details in the 'Methods' section). As shown in the FESEM image, the TiO_2 _NPs were well interconnected with one another, forming a rigid film that is strongly attached to the FTO glass substrate. The thickness of the TiO_2 _NP film was approximately 16.5 μm.

**Figure 3:**
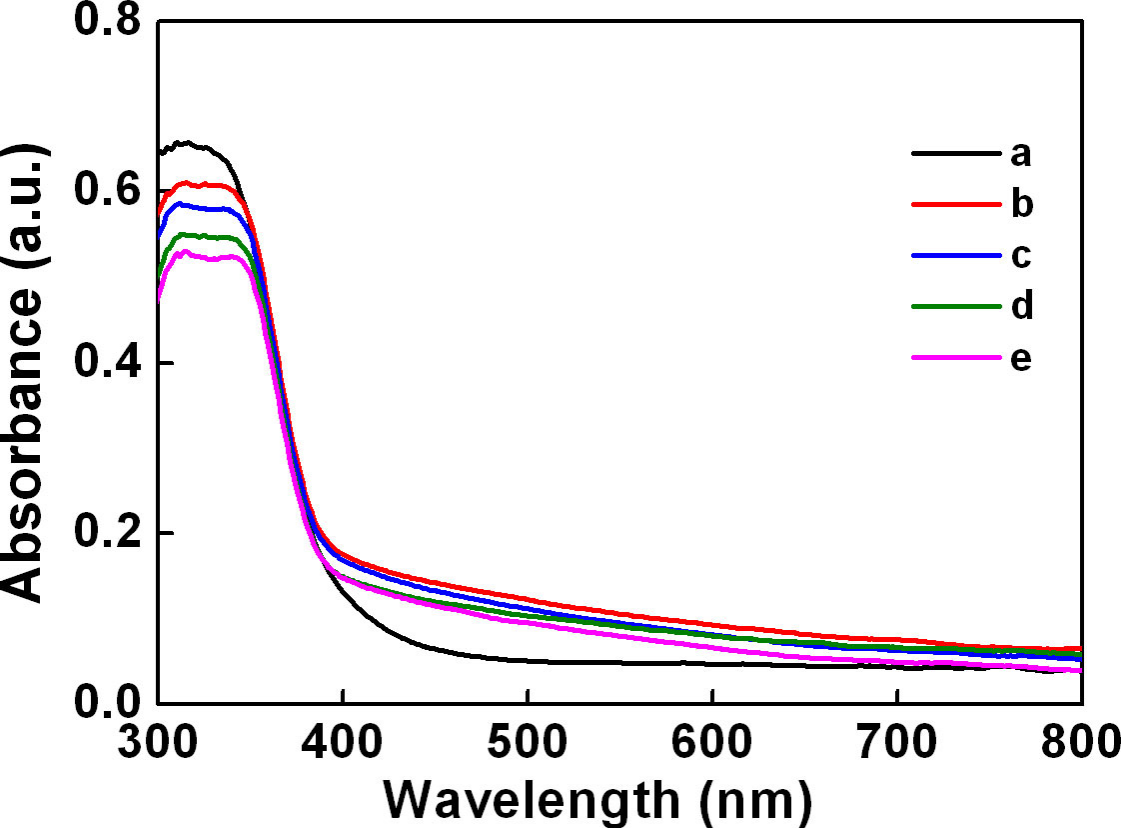
**Diffuse reflectance UV-VIS spectra**. (a) TiO_2 _and (b) PCBM/TiO_2_. PCBM/TiO_2 _irradiated at (c) 3.6 × 10^16^, (d) 7.2 × 10^16^, and (e) 1.44 × 10^17 ^cm^-2^.

**Figure 4:**
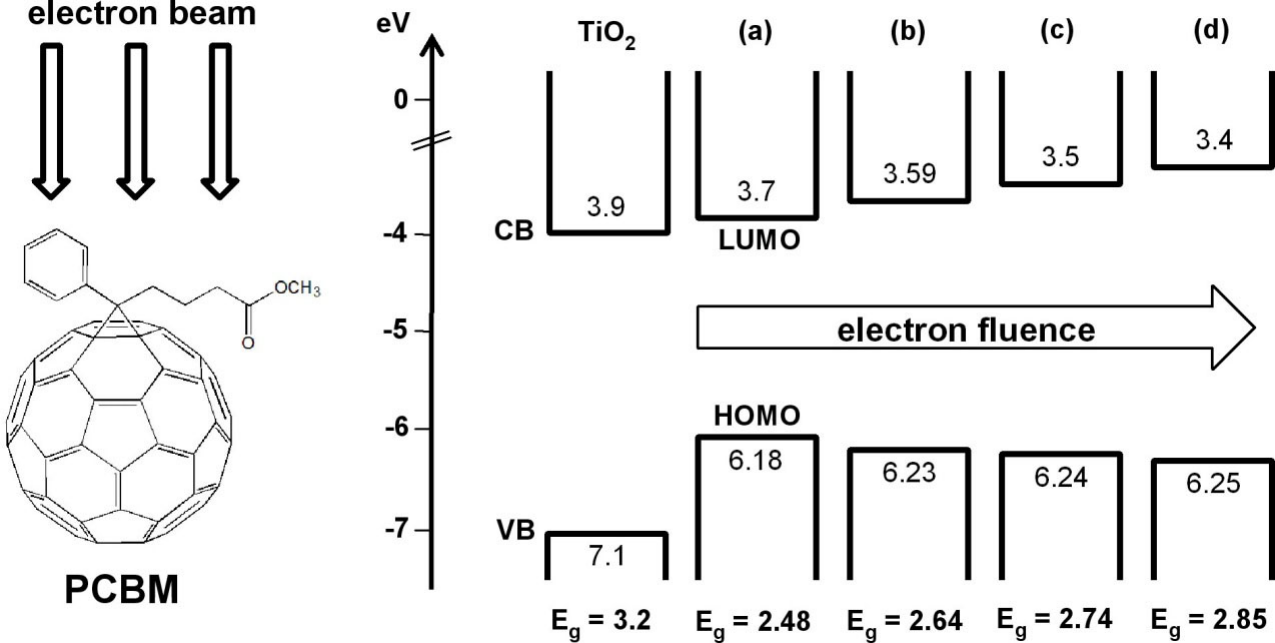
**Band structure of PCBM after electron beam irradiation of different fluences**. (a) Non-irradiated PCBM. (b) Irradiated PCBM at 3.6 × 10^16^, (c) 7.2 × 10^16^, and (d) 1.44 × 10^17 ^cm^-2^.

We observed that transparent TiO_2 _NP film became slightly yellowish after the PCBM coating. The UV-VIS absorption spectra shown in Figure 3 more clearly characterize the optical properties of the TiO_2 _NP films. When PCBM was coated on TiO_2_, visible light absorption of TiO_2 _in the wavelength range of 390 to 800 nm was increased, while absorption of UV in the range of 300 to 360 nm was decreased. In addition, when PCBM/TiO_2 _was irradiated with an electron beam, the absorbance in both UV and visible light region decreased gradually as the electron fluence increased. In our previous work, we reported that the bandgap of electron-irradiated PCBM increased as the electron fluence was increased. The modification of the bandgap was attributed to the change in the molecular structure of PCBM by electron irradiation. From these facts, we could conclude that the effective bandgap of electron-irradiated PCBM/TiO_2 _also increased as the electron fluence increased (Figure 4).

**Figure 5:**
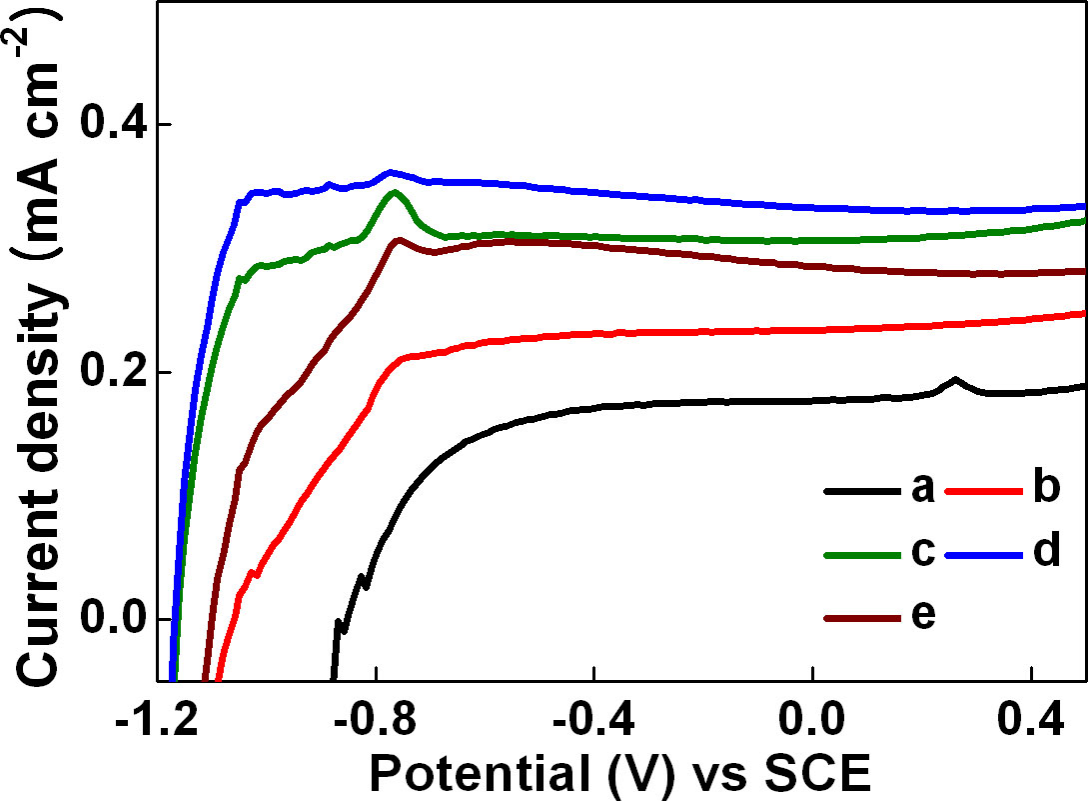
**Current density-potential curves of TiO_2 _and PCBM/TiO_2 _electrodes irradiated at different electron fluences under illumination**. (a) Non-irradiated TiO_2 _and (b) PCBM/TiO_2_. PCBM/TiO_2 _irradiated at (c) 3.6 × 10^16^, (d) 7.2 × 10^16^, and (e) 1.44 × 10^17 ^cm^-2^.

In order to investigate the band-tuning effect caused by the electron irradiation, we tried to characterize the PEC cell device performances using the electron-irradiated PCBM/TiO_2 _electrodes. The measurement results of the PEC responses of bare TiO_2_, PCBM/TiO_2_, and electron-irradiated PCBM/TiO_2 _electrodes are listed on Table 1, and the typical current density-potential curves of the electrodes are shown in Figure 5. The saturated current density at 0 V vs. saturated calomel electrode under dark conditions of all the electrodes was less than 15 μA cm^-2^. Under illumination of simulated solar light, bare TiO_2 _nanoparticle electrode shows saturated photocurrent density (*J*_ph_) of 176 μA cm^-2 ^and open-circuit potential (*E*_ocp_) of -0.85 V vs. SCE. After coating PCBM on TiO_2 _nanoparticles, the PEC performance was improved: *J*_ph _and *E*_ocp _of PCBM/TiO_2 _electrode increased to 234 μA cm^-2 ^and -1.05 V vs. SCE, respectively. The improvement in *J*_ph _and *E*_ocp _is attributed to the increment of visible light absorption of PCBM compared to that of TiO_2_. After electron irradiation of PCBM/TiO_2 _electrode at electron fluence of 3.6 × 10^16 ^cm^-2^, *J*_ph _and *E*_ocp _increased from 234 to 306 μA cm^-2 ^and -1.05 to -1.16 V vs. SCE, respectively. The PEC performance of PCBM/TiO_2 _electrode was further improved through electron irradiation of increased electron fluence. Both *J*_ph _and *E*_ocp _of electron-irradiated PCBM/TiO_2 _were increased with increasing the electron fluence. *J*_ph _increased to 333 μA cm^-2^, and *E*_ocp _increased to -1.16 V vs. SCE at the electron fluence of 7.2 × 10^16 ^cm^-2^.

**Table 1:** Photoelectrochemical performance of various electrodes investigated

	*J*_ph _(μA cm^-2^)	*E*_ocp _(V) vs. SCE
TiO_2_	176	-0.85
PCBM/TiO_2_	234	-1.05
PCBM/TiO_2_ (3.6 × 10^16 ^cm^-2^)	306	-1.16
PCBM/TiO_2_ (7.2 × 10^16 ^cm^-2^)	333	-1.16
PCBM/TiO_2_ (1.44 × 10^17 ^cm^-2^)	285	-1.10

The fact that the PEC performance of PCBM/TiO_2 _electrode was improved by electron fluence is interesting because electron irradiation increases the bandgap of PCBM and accordingly decreases the light absorption. As verified in our previous work, the LUMO level of PCBM shifts upward to the vacuum energy level as electron fluence increases. Since the bandgap of PCBM is much lower than that of TiO_2_, electron-hole pairs produced in PCBM can contribute to the increase in the photo-current of TiO_2_. However, the energy difference between the LUMO energy level of PCBM and the conduction band edge minimum of pure TiO_2 _is 0.2 eV, which might not be high enough for efficient electron transfer from PCBM to TiO_2 _[29]. Since LUMO energy level of PCBM is up-shifted by electron irradiation, electron-irradiated PCBM provides higher driving force of electron injection from PCBM to TiO_2 _[25]. This can explain why *J*_ph _of electron-irradiated PCBM/TiO_2 _electrodes was increased by increasing the electron fluence. Moreover, the increase in the energy difference between the LUMO energy level of electron-irradiated PCBM and the conduction band edge minimum of TiO_2 _provides efficient charge separation of the photo-excited electron-hole pairs, thereby improving *E*_ocp _[30].

However, when the electron fluence was further increased to 1.44 × 10^17 ^cm^-2^, the PEC performance of electron-irradiated PCBM/TiO_2 _became worse. As shown in Figure 4, the LUMO energy level of PCBM was constantly up-shifted toward the vacuum energy level as the electron fluence was increased. The up-shift in the LUMO energy level of electron-irradiated PCBM increases the driving force of electron injection from PCBM to TiO_2_. With the up-shift in the LUMO energy level, the bandgap of the electron-irradiated PCBM also increases with increasing the electron fluence. The increase in the bandgap reduces the light absorption of PCBM and consequently deteriorates the PEC performance. Therefore, electron irradiation induces the two contradictory effects on the PEC performance of the electron-irradiated PCBM/TiO_2_, and this suggests that there is an optimum electron fluence at which the PEC performance is maximized. In our experiments, *J*_ph _increased by approximately 90% and *E*_ocp _increased by approximately 36% compared to bare TiO_2 _at an optimum electron fluence at 7.2 × 10^16 ^cm^-2^.

## Conclusions

Using the fact that the electronic band structure of PCBM can be modified by electron irradiation, PCBM/TiO_2 _electrodes were fabricated and tested in a PEC cell. We observed that electron irradiation on PCBM/TiO_2 _electrodes led to an increase in *J*_ph _by approximately 90% and *E*_ocp _by approximately 36% at an optimum electron irradiation condition. These results show that electron irradiation approach can be a good tool to tune the bandgap and the band-edge positions of PCBM and provide an evidence that the approach is useful for PEC device application. We believe that the electron irradiation strategy can also control the electronic band structures of other organic semiconducting materials, and thus, this strategy can improve the performances of PEC and photocatalytic devices.

## Competing interests

The authors declare that they have no competing interests.

## Authors' contributions

The work was carried out by the collaboration between all authors. SOC initiated the idea of electron irradiation on PCBM/TiO_2 _electrodes. SHY performed the electron irradiation experiments. SHY and GA performed the construction of PEC cell and measurement of PEC responses of electron-irradiated PCBM/TiO_2 _electrodes. JMK and SHH carried out the diffuse reflectance UV-VIS spectroscopy measurements of electron-irradiated PCBM/TiO_2_. SOC and SHY analyzed the data and suggested the mechanism of improvement of electron-irradiated PCBM/TiO_2 _electrodes. All authors read and approved the final manuscript.
